# An Integrated Method to Analyze Farm Vulnerability to Climatic and Economic Variability According to Farm Configurations and Farmers’ Adaptations

**DOI:** 10.3389/fpls.2017.01483

**Published:** 2017-08-29

**Authors:** Guillaume Martin, Marie-Angélina Magne, Magali San Cristobal

**Affiliations:** ^1^AGIR, Université de Toulouse, INRA, INPT, INP-EI PURPAN, ENSFEA Castanet-Tolosan, France; ^2^GenPhySE, Université de Toulouse, INRA, INPT, INP-ENVT Castanet-Tolosan, France; ^3^INRA, UMR 1201 Dynafor Castanet-Tolosan, France

**Keywords:** longitudinal analysis, resilience, adaptive capacity, farm management, livestock system, linear mixed models

## Abstract

The need to adapt to decrease farm vulnerability to adverse contextual events has been extensively discussed on a theoretical basis. We developed an integrated and operational method to assess farm vulnerability to multiple and interacting contextual changes and explain how this vulnerability can best be reduced according to farm configurations and farmers’ technical adaptations over time. Our method considers farm vulnerability as a function of the raw measurements of vulnerability variables (e.g., economic efficiency of production), the slope of the linear regression of these measurements over time, and the residuals of this linear regression. The last two are extracted from linear mixed models considering a random regression coefficient (an intercept common to all farms), a global trend (a slope common to all farms), a random deviation from the general mean for each farm, and a random deviation from the general trend for each farm. Among all possible combinations, the lowest farm vulnerability is obtained through a combination of high values of measurements, a stable or increasing trend and low variability for all vulnerability variables considered. Our method enables relating the measurements, trends and residuals of vulnerability variables to explanatory variables that illustrate farm exposure to climatic and economic variability, initial farm configurations and farmers’ technical adaptations over time. We applied our method to 19 cattle (beef, dairy, and mixed) farms over the period 2008–2013. Selected vulnerability variables, i.e., farm productivity and economic efficiency, varied greatly among cattle farms and across years, with means ranging from 43.0 to 270.0 kg protein/ha and 29.4–66.0% efficiency, respectively. No farm had a high level, stable or increasing trend and low residuals for both farm productivity and economic efficiency of production. Thus, the least vulnerable farms represented a compromise among measurement value, trend, and variability of both performances. No specific combination of farmers’ practices emerged for reducing cattle farm vulnerability to climatic and economic variability. In the least vulnerable farms, the practices implemented (stocking rate, input use…) were more consistent with the objective of developing the properties targeted (efficiency, robustness…). Our method can be used to support farmers with sector-specific and local insights about most promising farm adaptations.

## Introduction

The biophysical, economic and social context of agricultural production is increasingly unpredictable and volatile (Thompson and Scoones, 2009), driven by complex and interrelated contextual changes, including scarcity of natural resources, climate change, increasing food demand, and administrative regulation. Along with changes in trends, climatic and economic variability, i.e., variation around long-term means, is also growing (Wright, 2011; IPCC, 2013). In a changing context, the primary and immediate effects on nature and society result from variability rather than from means (Katz and Brown, 1992). Consequently, farmers must continuously adapt to reduce their vulnerability to climatic and economic variability (Howden et al., 2007; van Vuuren et al., 2011).

The vulnerability of any system (at any scale) is considered a function of exposure and sensitivity of that system to a range of hazards and the adaptive capacity of the system to cope with, adapt to, or recover from the effects of these conditions (Smit and Wandel, 2006). More precisely, exposure usually refers to the duration, extent and frequency of climatic and economic perturbations influencing the system (Adger, 2006). Sensitivity is the degree to which the system responds to such perturbations (Gallopín, 2006). Exposure and sensitivity determine the potential impact that occurs given the climatic and economic variability. Adaptive capacity is the degree to which a system can adjust to, moderate, or offset the potential impacts, or take advantage of opportunities created by a given climatic or economic event (Schneider et al., 2001). Actual impact is the impact that remains after accounting for adaptive capacity, especially adaptations implemented by system managers confronted with climatic and economic variability.

The need to adapt to decrease farm vulnerability to adverse contextual events has been extensively discussed on a theoretical basis (Meinke et al., 2009; Darnhofer et al., 2010b). Farmers now need sector-specific and local insights about adaptations to decrease farm vulnerability to climatic and economic variability. Research has developed two types of approaches to address this issue: model-based studies and field-based studies for *ex ante* and *ex post* analysis, respectively.

Model-based studies are interesting in that they enable strict application of the vulnerability framework by isolating farm sensitivity from adaptive capacity: *ex ante* simulations can be run without considering farmers’ adaptations. Still, model-based studies have several drawbacks, including (i) limited ability to reproduce farm management and production when applied to new sites (Ewert et al., 1999; Palosuo et al., 2011) and (ii) simulation of farm adaptations in a necessarily limited and simplified context that ignores key issues to farmers (e.g., family issues). These drawbacks compromise the relevance for farmers of adaptation recommendations originating from model-based studies.

Field-based studies have the potential to address farm adaptation and consequences of farm vulnerability in the multifactorial context farmers encounter (Nicholas and Durham, 2012). However, they do not allow breaking down vulnerability into exposure, sensitivity, and adaptive capacity. Sensitivity is not measurable in the field, since farmers often react to adverse events by implementing adaptations. Since it is a potential, adaptive capacity cannot be assessed exhaustively, unlike adaptations implemented by farmers. Thus, it is the actual impact of climatic and economic variability that is most easily assessed according to farm configurations and farmers’ adaptations.

Field-based studies are seldom reported, and examples of them have several drawbacks. Many studies are limited to a single driver of contextual change, mainly climate (Reidsma et al., 2009; Dong et al., 2015). Farm vulnerability is often reduced to productivity or income issues by measuring changes in yield or income over several years (Reidsma et al., 2010; Dong et al., 2015), whereas technical efficiency issues (and related environmental and economic impacts) are often neglected, yet are key to sustainable agriculture (Keating et al., 2010). Many studies focus on strategic changes and consequently use multi-year time steps [from 5 years in Rueff et al. (2012) to 14 years in García-Martínez et al. (2009)], whereas year-to-year tactical adaptations can have significant impacts on farm performances and related vulnerability. Nearly all studies (Nicholas and Durham, 2012) fail to address interactions among adaptation options (i.e., co-occurrence of changes in farmers’ practices) over time, possible delayed impacts of their implementation, and resulting trade-offs in farm performances that explain vulnerability (Reed et al., 2013).

There is a need to develop an integrated method that enables the assessment of farm vulnerability to multiple and interacting contextual change drivers (both climatic and economic) and to explain how this vulnerability can be reduced according to farm configurations and farmers’ technical adaptations over time. This is precisely the purpose of this study. To this end, we assume that considering not only the mean level but also the trend and variability of farm performances (e.g., farm productivity, farm economic efficiency) is required to assess farm vulnerability. We have developed such a generic (applicable to all farm types) method and applied it to cattle farms. Cattle farms are interesting to illustrate the merits of longitudinal analysis, since animal health and production in a given year can be strongly influenced by fodder and grain production of the previous year. Results of this analysis are presented and discussed, along with the relevance and applicability of the method.

## Materials and Methods

### Overview of the Statistical Method

**FIGURE 1:**
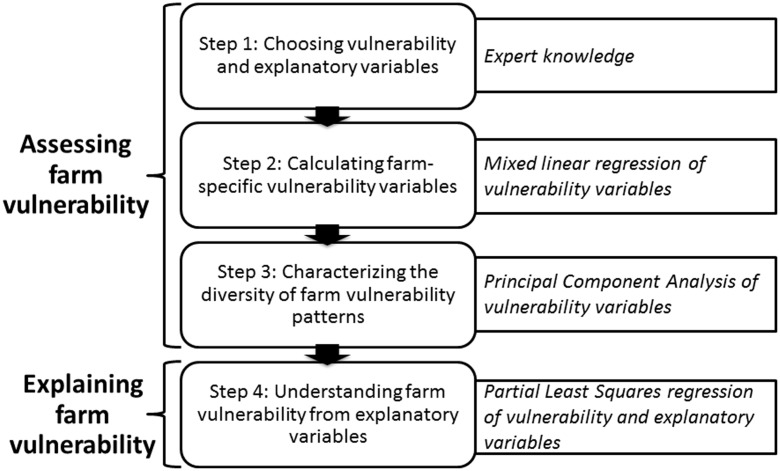
Step-by-step representation of the method. Knowledge and statistical tools used for each step are given in italics.

Our method (**Figure 1**) has two main objectives: (i) assessing farm vulnerability to the drivers of multiple contextual changes and (ii) explaining this vulnerability as a function of initial farm configurations and farmers’ multiple technical adaptations. To assess farm vulnerability to climatic and economic variability, a naive approach would consist of calculating the empirical variance of vulnerability variables for a farm over a period of time, i.e., the mean of the deviations of these vulnerability variables from their empirical mean. In doing so, however, the longitudinal aspect and the common trend would be lost, whereas farm vulnerability to climatic and economic variability can vary over time according to farmers’ adaptations. A farm with stable or increasing vulnerability variables over the study period maintains or reduces its vulnerability over time. The idea is thus to consider a trend for each vulnerability variable per farm and the deviations from this trend.

**FIGURE 2:**
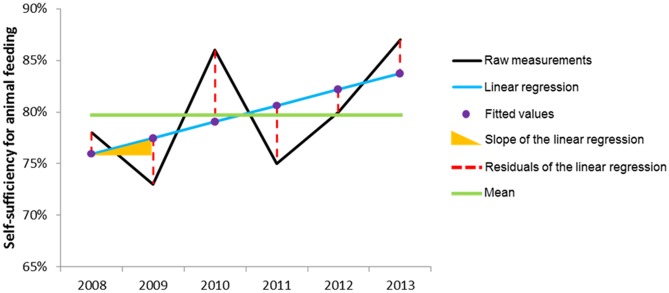
Overview of the three types of vulnerability variables to assess farm vulnerability (restricted to self-sufficiency for animal feeding in this figure) to climatic and economic variability: raw measurements (fitted or mean values can be used), slope of the linear regression over time (general trend over the period), and residuals of the regression (as a measure of stability and robustness).

Following this idea, our method considers farm vulnerability as a function of the raw measurements (or fitted values, or mean level over the entire period) of vulnerability variables, the slope of the linear regression of these measurements over time, and the residuals of this linear regression (**Figure 2**). These three mathematical parameters describe the mean level, trend, and variability of each vulnerability variable, respectively. Among all possible combinations, the lowest farm vulnerability to climatic and economic variability is obtained through a combination of high values of measurements (i.e., toward “good” mean performances), a stable or increasing trend (i.e., toward improvement), and low variability (i.e., toward stability and robustness) for all vulnerability variables considered. In contrast, the highest farm vulnerability results from a combination of low measurements (i.e., poor mean performances), a decreasing trend (i.e., toward decline) and high variability (i.e., toward instability) for all vulnerability variables considered. Our method then relates multiple farm vulnerability variables to explanatory variables that illustrate farm exposure to climatic and economic variability and initial farm configurations and farmers’ technical adaptations over time.

### Steps of the Statistical Method

Step 1: Choosing vulnerability and explanatory variables.

Variable choice involves selecting a set of vulnerability variables (**Table 1A**) that characterize cattle farm vulnerability and a set of explanatory variables (**Table 1A**) that characterize cattle farm exposure to climatic and economic variability, initial farm configurations, and farmers’ technical adaptations over time to climatic and economic variability. If needed, variables can be transformed.

**Table 1A:** Vulnerability variables describing individual farms.

Category	Sub-category	Variable	Abbreviation	Unit
Vulnerability	Productivity	Farm productivity	Y.Prod	kg protein/ha/year
		Slope of the linear regression of Prod	Sl.Prod	kg protein/ha/year
		Residuals of the linear regression of Prod	R.Prod	kg protein/ha/year
	Economic efficiency	Economic efficiency of production	Y.EconEff	%/year
		Slope of the linear regression of EconEff	Sl.EconEff	%/year
		Residuals of the linear regression of EconEff	R.EconEff	%/year


Step 2: Calculating farm-specific vulnerability variables.

Regression is performed for each vulnerability variable as a function of the year to extract the trend and the residuals. This must be achieved for all farms, not only surveyed farms. Surveyed farms can be viewed as a random sample of farms from a much larger set. This sampling is accounted for in the statistical analysis using linear mixed models. A random-coefficient regression provides the overall mean (an intercept common to all farms), overall trend (a slope common to all farms), a random deviation from the overall mean for each farm, and a random deviation from the overall trend for each farm. Residuals of the random regression provide additional measures of stability. Numerically, the effects of farms as deviations from the overall mean and overall slope decrease to 0 when considered as random effects rather than fixed effects. Therefore, extreme values become less extreme in a random-regression model, which avoids over-emphasizing outliers. Analysis is performed using the statistical package lme4 (Bates et al., 2015) in R software (R Development Core Team, 2015).

The random regression is written as:

$$y_{i,j,t}=\alpha +\beta t+\alpha_{j}+\beta_{j}t+a_{i}+b_{i}t+e_{i,j,t}$$

where *y*_i,j,t_ is the measure of the vulnerability variable for farm *i* in year *t* for level *j* of a certain combination of cofactors (e.g., farm type: beef, dairy, or mixed),

α is the mean level of the vulnerability variable at time 0 (beginning of the study period),

β is the mean trend of the vulnerability variable over the period,

α_j_ is the mean level of the vulnerability variable at time 0 specific to a combination *j* of cofactors,

β_j_ is the mean trend of the vulnerability variable over the period specific to a combination *j* of cofactors,

*a*_i_ is the random effect of farm *i* on the level of the vulnerability variable at time 0; it is seen as a deviation from the overall mean α + α_j_ for farm *i*. All *a*_i_ are assumed to be independent and identically distributed (iid) $a_{i}∼N(0,\sigma_{a}^{2}),$

*b*_i_ is the random slope for farm *i*, the deviation from the overall trend β + β_j_. All *b*_i_ are assumed to be iid $b_{i}∼N(0,\sigma_{b}^{2}),$

e_i,t_ is the residual, assumed to be iid $e_{i,t}∼N\left(0,\sigma_{e}^{2}\right)$ and independent of all *a*_i_ and *b*_i_.

A correlation ρ_ab_ between the two random effects relative to the farm is assumed: ${\left(\begin{array}{c} a\\ b \end{array}\right)}_{i}∼N_{2}\,\;\left((\begin{array}{c} 0\\ 0 \end{array}),\left(\begin{array}{cc} \sigma_{a}^{2} & \rho_{ab}\sigma_{a}\sigma_{b}\\ \rho_{ab}\sigma_{a}\sigma_{b} & \sigma_{b}^{2} \end{array}\right)\right)$.

The information contained in *y*_i,t_ for *i* = 1,…*I* and *t* = 1,…*T* is partitioned into the prediction (fitted value) y^i,t=α+βt+αj+βjt+ai+bit and the residual *e*_i,t_.

As a direct consequence of model (1), each farm *i* is characterized by three aspects corresponding to (i) its level *a*_i_ at the beginning of the period expressed, i.e., the intercept, as a deviation from the overall performance level of all farms; (ii) its trend *b*_i_ over the period, as a deviation from the overall trend; and (iii) the variability $\sigma_{e,i}^{2}=\frac{1}{T}\Sigma_{t=1}^{T}e_{i,t}^{2}$ around the expected trajectory α + β*t* + α_j_ + β_j_*t* + *a*_i_ + *b*_i_*t*.

We used this model to study detailed measures for each farm at each point in time. The level was estimated either by a_i_, the fitted value at time 0 (intercept), the fitted value at a given time (e.g., middle of the period), or the mean level of vulnerability variable, raw values, or fitted values over the entire period (the last one representing de-noised data).

Step 3: Characterizing the diversity of farm vulnerability patterns.

Descriptive analysis of farm vulnerability consists of principal component analysis (PCA) of all vulnerability variables (raw measurements, slope and residuals of their linear regression each time). It identifies relationships among vulnerability variables and highlights their similarities and differences through correlations. It also distinguishes patterns of farms’ vulnerability trajectories. This makes it possible to verify whether some individuals display high (high average level, stable or increasing trend and low variability of vulnerability variables) or low vulnerability according to our hypotheses (see Overview of the Statistical Method). PCA is performed using the statistical package mixOmics (Lê Cao et al., 2009; González et al., 2011) in R software.

Step 4: Understanding farm vulnerability from explanatory variables.

Tools commonly used to select linear models sometimes have difficulties choosing a subset of explanatory variables. In the context of prediction, or when explanatory variables are numerous and highly correlated, it is often useful to use statistical tools to reduce the number of dimensions. Regression on principal components (after a PCA of all explanatory variables) can drastically reduce the number of explanatory variables in the model. However, principal components are not necessarily related to the response variable. This was the aim of developing the partial least squares (PLS) regression: to reduce dimensionality, use orthogonal explanatory variables, and search for the maximum covariance between response and explanatory variables.

Partial least squares is a multivariate projection-based method offering a compromise between PCA and linear modeling (Tenenhaus, 1998; Wold et al., 2001). Unlike linear modeling, it is not limited to a single response variable and unlike PCA, it can distinguish among sets of response and explanatory variables. The regression mode of PLS analysis consists of modeling explanatory relationships between variables of two datasets, i.e., PLS predicts multiple vulnerability variables from two types of explanatory variables (climatic and economic exposure variables) and variables describing initial farm configurations and farmers’ technical adaptations over time. PLS regression is performed using the statistical package mixOmics (Lê Cao et al., 2009; González et al., 2011) in R software.

### Case Study Application

#### Case Study Farms

**FIGURE 3:**
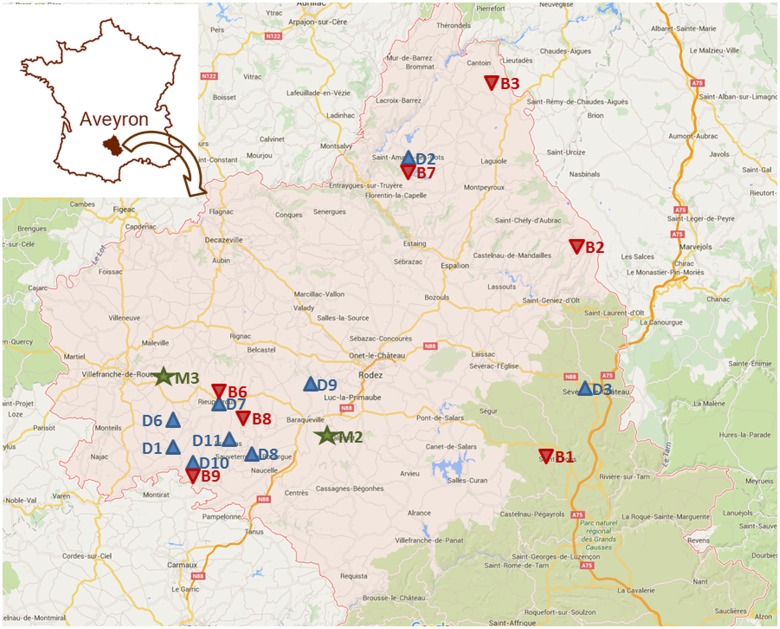
Map of the Aveyron department and location of the case study cattle farms: 9 (D)airy (blue), 2 (M)ixed (green) and 8 (B)eef (red). (Map data © 2015 Google; no further permissions are required for the use and reproduction of this image).

**FIGURE 4:**
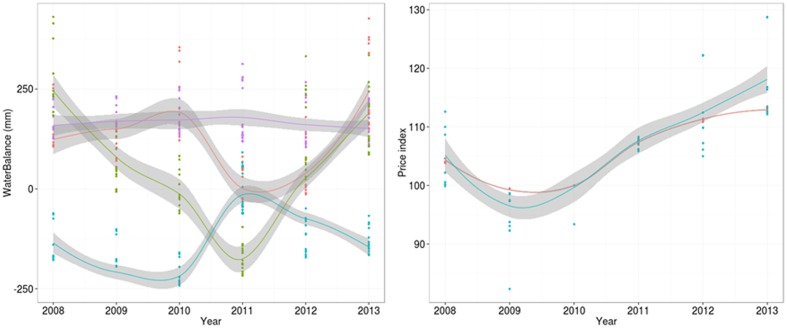
Dynamics of **(left)** seasonal water balances (red: autumn, green: spring, blue: summer, purple: winter) and **(right)** prices (red: inputs, blue: outputs) during the survey period. Gray areas represent smoothed areas (equal to one standard deviation) computed by loess around each line.

Our study was performed in the French department of Aveyron (**Figure 3**). At this regional scale, exposure to economic hazards is considered equal among cattle farm types (dairy, beef, mixed) but variable over time (**Figure 4**). In contrast, exposure to climatic hazards varies both among farms and over time (**Figure 4**). Aveyron contains a range of climatic conditions, from dry lowland plains to cold and snowy mountains. As a result, it has a wide diversity of cattle farms, from highly productive maize-based systems in lowland plains to less-productive grass-based systems in the mountains. We hypothesized that farm diversity was related to the diversity of initial farm configurations and farmers’ technical adaptations to climatic and economic variability.

The Chamber of Agriculture in Aveyron created a network of 24 beef and dairy cattle farms that are surveyed every year. Data are collected on the key aspects of livestock systems: geographic location, land use, crop and grassland yields, herd structure, animal production, animal feeding, labor requirements, and economic returns and costs. Surveys covered the period 2008–2013 and lasted 3–6 years, depending on the farm. We restricted analysis to 19 farms involved in at least 4 years of the survey (**Figure 3**). In the following text, farms are denoted by a letter (B: beef, D: dairy, M: mixed) followed by a number corresponding to the farm. We supplemented these surveys with downscaled weather data (8 km × 8 km grid), which were needed to calculate climatic indicators and two price indices (**Table 1B**).

**Table 1B:** Explanatory variables describing individual farms.

Category	Sub-category	Variable	Abbreviation	Unit
Exposure	Climate	Number of days with heat stress	HeatStress	day
		Earliness of the growing season	Earliness	°C-day
		Water deficit or excess in autumn	WaterAutumn	mm
		Water deficit or excess in summer	WaterSummer	mm
		Water deficit or excess in winter	WaterWinter	mm
		Water deficit or excess in spring	WaterSpring	mm
	Economics	Input price index	InputPrice	
		Output price index	OutputPrice	
Adaptive capacity: farmers’ management practices	Land use	Stocking rate	StockingRate	LU/ha
		Percentage of semi-natural pastures on the farm	%NatPast	%
		Percentage of grass-based ley pastures on the farm	%GrassPast	%
		Percentage of legume-based ley pastures on the farm	%LegPast	%
		Percentage of cropland on the farm	%Crop	%
		Percentage of cover crops on the farm	%CoverCrop	%
		Shannon index of diversity of the farmland	ShannonLand	/
		Amount of irrigation water used	IrrigWater	L/ha
		Mineral fertilization rate	NMinFert	kg N/ha
	Herd management	Spread of calving within the herd	CalvingSpread	month^-1^
		Replacement rate within the herd	ReplaceRate	%
		Shannon index of diversity of the herd	ShannonHerd	/
		Number of animal diets fed outside the farm	OffFarmDiets	diet
		Percentage of silage in animal diets	%SilageFeed	%
		Amount of fodder distributed per animal	FodderDistrib	t DM/LU
		Amount of concentrate distributed per animal	ConcDistrib	t DM/LU


#### Choice of Vulnerability and Explanatory Variables for the Cattle-Farm Case Study

The choice of vulnerability and explanatory variables relied on conceptual and indicator frameworks on adaptiveness of agricultural and socio-ecological systems (Darnhofer et al., 2010a; Biggs et al., 2012; Cabell and Oelofse, 2012). Following these frameworks, we measured two vulnerability variables that characterize vulnerability of cattle farms confronted with climatic and economic variability: the ability to remain (i) productive (based on internal feed resources) and (ii) technically and economically efficient (Darnhofer et al., 2010a; Cabell and Oelofse, 2012) (**Table 1A**). The two vulnerability variables measured were:

1. Farm productivity based on feed internal resources, i.e., the amount of protein in milk and meat produced per year and per hectare on the farm allowed by the amount of animal feed produced on the farm over the entire animal feed consumption (on a dry matter basis) to consider farm self-sufficient productivity:
  Farm productivity = production of protein in milk and meat (kg protein per year) × self-sufficiency for animal feeding/usable agricultural area (ha) and self-sufficiency for animal feeding = concentrate and forage produced on-farm (kg DM per year)/total concentrate and forage consumed by the herd (kg DM per year).
2. Economic efficiency of production, i.e., net economic output produced per unit of gross output:

Economic efficiency of production = (gross product - operational costs)/gross product

The two vulnerability variables were broken down into three aspects: raw measurements, slope of the linear regression of these measurements over time, and residuals of the regression.

We chose eight explanatory variables to illustrate cattle-farm exposure to climatic and economic variability (**Table 1B**). Exposure to climatic variability was assessed with the following six variables:

1. Earliness of the growing season, i.e., sum of degree days from 1 February to 1 of April of each year;
2. Heat stress on crop and forage plants, i.e., the number of days with a mean temperature above 25°C per year;
3. Water deficit or excess in spring, summer, autumn, and winter, respectively, i.e., the difference between rainfall and evapotranspiration during each season of each year.

Exposure to economic variability was assessed with the following two variables:

1. Integrated input price index for each year (IDELE, 2015) integrating the variability of prices of seeds, fertilizers, pesticides, feedstuffs, drugs, energy, machinery and the cost of its maintenance;
2. Integrated output price index for each year (INSEE, 2015) integrating the variability of prices of milk and meat.

We selected explanatory variables to illustrate farm configurations and farmers’ technical adaptations over time (**Table 1B**). All of the conceptual and indicator frameworks cited above suggest that greater diversity [i.e., variety, balance, and disparity, as defined by Biggs et al. (2012)] promotes redundancy within such systems and increases their adaptive capacity. These frameworks also suggest that adaptive capacity is promoted by greater internal recycling of waste (e.g., manure), which is promoted by connectivity among system components. Accordingly, we selected the following 16 variables to illustrate land use and herd-management practices employed by farmers on cattle farms:

a. Land use:
  a. stocking rate, i.e., number of animals per unit of farm area
  b. percentages of semi-natural pastures, grass-based ley pastures, legume-based ley pastures and cropland in the farmland area
  c. percentage of cover crops in the farmland area
  d. shannon index of diversity of the farmland area, i.e., the more diverse the land-use types on the farm, the higher the index
  e. amount of irrigation water used per unit of farm area
  f. mineral fertilization rate per unit of farm area.
b. Herd management:
  a. spread of calving within the herd, calculated as the inverse of the calving duration in months, i.e., the more grouped the calving, the higher the index
  b. replacement rate within the herd
  c. shannon index of diversity of the herd, i.e., the diversity of the age and sex classes of cattle on the farm;
  d. number of animal-days of off-farm grazing/feeding
  e. percentage of maize and fodder silage in animal diets on a dry matter basis
  f. amount of fodder distributed per animal
  g. amount of concentrate distributed per animal.

## Results

### Characterization of Cattle Farm Vulnerability to Climatic and Economic Variability

#### Overview of Variables of Cattle Farm Vulnerability

**FIGURE 5:**
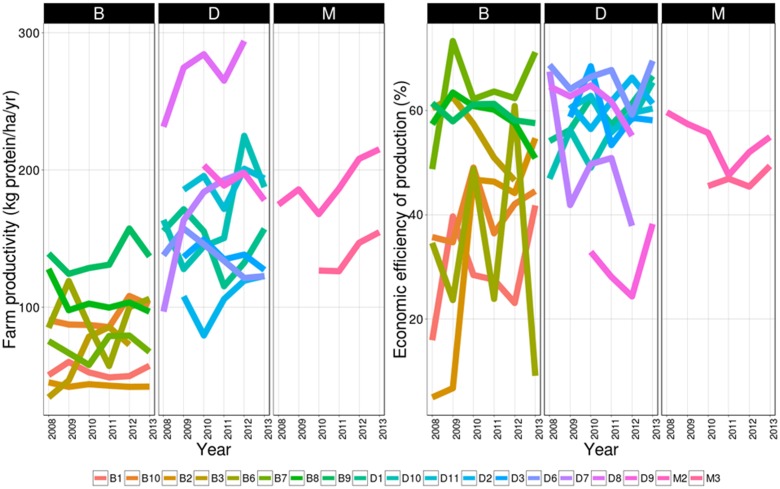
Dynamics of the two vulnerability variables, **(left)** farm productivity and **(right)** economic efficiency of production for (B)eef, (D)airy, and (M)ixed cattle farms during the survey period.

**FIGURE 6:**
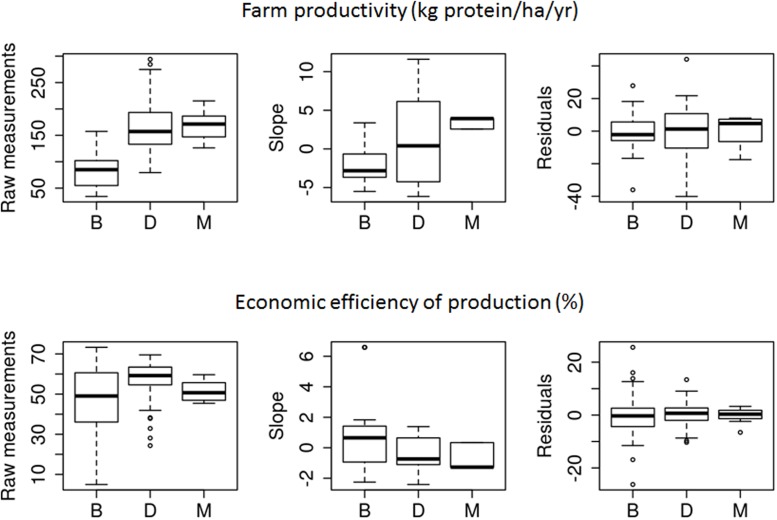
Distribution of the six vulnerability variables: raw measurements, slope, and residuals for both farm productivity and economic efficiency of production for (B)eef, (D)airy, and (M)ixed cattle farms over the survey period. Boxes represent the interquartile range containing 50% of values; the line across the boxes corresponds to the median value; whiskers are the highest and lowest values excluding outliers, i.e., cases with values up to 1.5 box lengths from the upper and lower edge of the box.

Farm productivity varied greatly among cattle farms, with means ranging from 43 kg protein/ha/year in B2, the least productive system, to 270 kg protein/ha/year in D8, the most productive system (**Figure 5**). ANOVAs showed that overall levels and trends of farm productivity differed significantly among farm types (*P* < 0.001 and *P* < 0.001, respectively; **Figures 5**, **6**). Dairy systems tended to have mean farm productivity levels (167.7 kg protein/ha/year) similar to those of mixed systems (169.3 kg protein/ha/year) and higher than those of beef systems (83.3 kg protein/ha/year). An improvement was observed in farm productivity in dairy (0.9 kg protein/ha/year) and mixed systems (3.4 kg protein/ha/year), whereas the trend declined in beef systems (-2.1 kg protein/ha/year) over the study period. However, trends differed significantly among farms, independent of farm type (*P* < 0.001). For instance, while D8 displayed a major improvement over the study period (6.8 kg protein/ha/year), D6 experienced a strong decline (-6.1 kg protein/ha/year). Regarding variability in farm productivity, among farm types, mixed systems (7.6 kg protein/ha/year) and beef systems (7.5 kg protein/ha/year) had lower mean absolute residuals than dairy systems (12.2 kg protein/ha/year), but these differences were not significant (*P* = 0.917). Independent of farm type, mean absolute residuals varied threefold, ranging from 5.2 kg protein/ha/year for D3 to 16 kg protein/ha/year for D7, but differences were also not significant (*P* = 1).

Economic efficiency of production varied greatly and differed significantly among farms independent of farm type (*P* < 0.001), with means ranging from 29.4%/year in B1, the least efficient system, to 66.0%/year in D6, the most efficient system (**Figures 5**, **6**). Differences were also significant among farm types (*P* = 0.002): dairy systems were the most efficient systems overall (56.8%), followed by mixed systems (51.5%/year) and beef systems (46.6%/year). Trends in economic efficiency also differed significantly among farm types (*P* < 0.012). A slight improvement was observed in beef systems (0.7%/year), compared to decreases in mixed systems (-0.6%/year) and dairy systems (-0.4%/year) over the study period. Likewise, significant differences were observed among farms, with trends (*P* < 0.001) ranging from -2.4%/year in D7 to 1.4%/year in D10 for dairy systems and from -2.3%/year in B3 to 6.6%/year in B7 for beef systems. When focusing on the variability in economic efficiency, differences among farm types were not significant (*P* = 0.901). Nonetheless, mean absolute residuals were nearly twice as high in beef systems (6.1%/year) as in dairy systems (3.3%/year) and were limited to 2.0%/year in mixed systems. Independent of farm type, between-farm differences were not significant (*P* = 1). Mean absolute residuals ranged from 0.9%/year in M3 to 14.7%/year in B6.

#### Relationships among Variables of Cattle-Farm Vulnerability

A random regression coefficient was fitted to each vulnerability variable, with an average effect of farm type (dairy, beef, mixed), a per-farm average random effect, an overall time effect of farm type, and a per-farm random effect of time. A variety of hypotheses were tested within the model. For farm productivity, farm type had a significant effect on the intercept (*P* = 0.002) but not on the slope. Year had a significant effect overall (*P* = 0.04). For economic efficiency, neither farm type nor year was significant. Therefore, the final models were as follows:

$${\mathrm{Pr}od}_{i,j,t}=\alpha +\beta t+\alpha_{j}+a_{i}+b_{i}t+e_{i,j,t}$$

$${EconEff}_{i,j,t}=\alpha +a_{i}+b_{i}t+e_{i,j,t}$$

**FIGURE 7:**
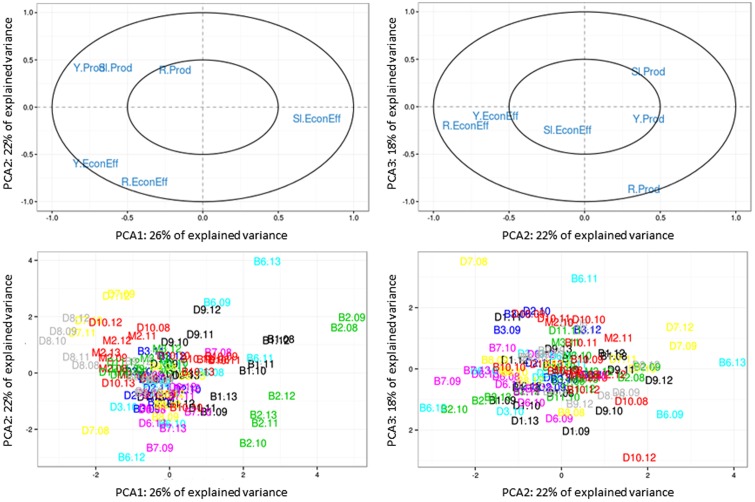
Principal component analysis (PCA) loading of the six vulnerability variables (raw measurement, trend, and variability of farm productivity and economic efficiency of production along components 1–3 (explaining 66% of the variance), showing **(top)** relationships of the variables along these dimensions and **(bottom)** individual data points denoted with farm numbers (B: beef, D: dairy, or M: mixed followed by a number corresponding to the farm) followed by numbers corresponding to each year. Full names of variables are provided in **Tables 1A,B**.

According to the Kaiser criterion, projecting the set of vulnerability variables into the PCA required analyzing three components, which explained 66% of the observed variance (**Figure 7**). The PCA showed that no cattle farm displayed low vulnerability to climatic and economic variability, i.e., a high level, a stable or increasing trend, and low variability for both farm productivity and economic efficiency. On the first component of the PCA (explaining 26% of the variance), the measure of farm productivity was positively correlated with the measure of economic efficiency (**Figure 7**). Among farms, this indicated that the most productive farms (i.e., producing the most protein per hectare using the least input of external feed) could also be the most economically efficient (i.e., producing the most per unit of input invested). It was also observed that the higher the level of farm productivity, the higher its slope over the study period. All the variables mentioned above were negatively correlated with the slope of economic efficiency. Farms with the highest levels of farm productivity and economic efficiency tended to have decreasing trends of economic efficiency. The farms that experienced improvement in farm productivity (positive slopes) tended to decline in economic efficiency. The second and third components (explaining 40% of the variance) were driven mostly by residuals of the linear regression of economic efficiency and farm productivity, respectively. Hence, the second PCA axis represented a gradient of interannual variability in economic efficiency, while the third axis described a gradient of interannual variability in farm productivity.

The distribution of individual data points (combinations of farm × year) in the PCA revealed contrasting patterns among farms. For instance, farms B2, B1, and B6 had low levels of farm productivity and economic efficiency, decreasing trends in farm productivity, improvement in economic efficiency, and high interannual variability. These farms were considered the most vulnerable in the sample. Conversely, farms D8, D7, and M2 were considered the least vulnerable in the sample. Analysis of all six vulnerability variables for individual farms indicated that, contrary to our hypothesis, no farm displayed a high level, a stable or increasing trend, and low residuals for both farm productivity and economic efficiency. For this reason, the least vulnerable farms represented a compromise between level, trend and variability of both performances.

### Cattle Farm Vulnerability According to Climatic and Economic Variability and Farmer Management

The *Q*^2^ criterion indicated that only the first component of the PLS could be considered. Based on graphical outputs of the analysis, we also included the second component. Explanatory variables were able to explain only one vulnerability variable in the first component, the level of farm productivity, and one in the second component, the slope of farm productivity. Vulnerability variables based on economic efficiency of production were not related to any explanatory variable.

**FIGURE 8:**
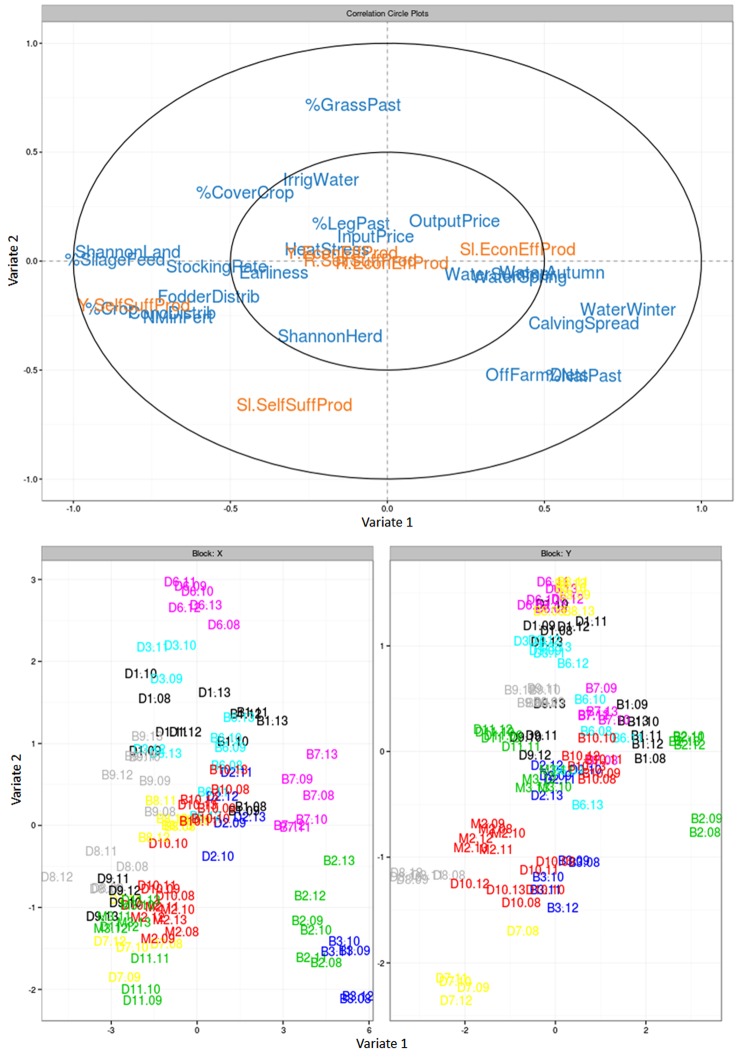
Partial least squares (PLS) regression of the six vulnerability variables (orange) according to exposure, farm configuration and farmers’ technical adaptation variables (blue) along components 1 and 2, showing **(top)** relationships of the variables along these dimensions and (bottom) individual data points **(bottom)** denoted with farm numbers (B: beef, D: dairy, or M: mixed followed by a number corresponding to the farm) followed by numbers corresponding to each year. Full names of variables are provided in **Tables 1A,B**.

In the first component of the PLS regression (**Figure 8**), the level of farm productivity was positively correlated with seven management-practice variables: percentage of cropland in the farmland, percentage of silage in animal diets, Shannon index of the farmland, amount of fodder and concentrate distributed per animal, stocking rate, and mineral nitrogen fertilization rate. It was negatively correlated with two management-practice variables, namely percentage of semi-natural grasslands in the farmland and spread of calving, and two exposure variables, namely excess water in autumn and winter. Thus, it appears that farms relying solely on semi-natural grasslands with grouped calving had lower farm productivity levels than diversified systems having higher stocking and mineral fertilization rates, higher amounts of feed distributed per animal (and consequently less grazing), and calving distributed throughout the year.

In the second component of the PLS regression, the slope of the linear regression of farm productivity was negatively correlated with the percentage of grass-based ley pastures in the farmland, the amount of irrigation water used, and the percentage of cover crops in the farmland. Thus, the farms displaying improvements in farm productivity over the study period had low percentages of grass-based ley pastures and cover crops and did not depend on irrigation water.

Analysis of the distribution of individual data points (combinations farm × year) in the PLS identified the most and least vulnerable farms and their position with respect to climatic and economic conditions, initial farm configurations, and farmers’ technical adaptations. Distribution of farms within the factorial design of the X block of the PLS (**Figure 8**) was mainly determined by farms’ initial positions. This indicates that adaptations implemented during the survey period did not compensate for initial differences in farm configuration and that no farm operated a substantial technical transition.

The most vulnerable farms (B2, B1, and B6) displayed poorly diversified farmlands compared to the least vulnerable farms (D7, D8, and M2) (mean Shannon index in the range 0.81–1.24 vs. 1.92–2.28, respectively, Appendix 1) that had a higher percentage of their area as cropland (31.9–42.4% vs. 3.4–18.7%). Farmland diversity distributes biomass production periods of fields over the year and reduces farm exposition to climatic hazards. The most vulnerable farms also tended to harvest less fodder in the form of silage (mean percentage in the range 13.4–53.8% vs. 76.9–80.1%, respectively) resulting in lower amounts of fodder distribution (1.66–2.58 t DM/LU vs. 3.22–3.95 t DM/LU, respectively). As compared to hay-making, silage harvesting provides flexibility against adverse weather conditions and therefore enables to adapt to the conditions of each year.

In both groups of farms, i.e., the least and most vulnerable, respectively, farms displayed contrasting individual patterns on the X block of the PLS beyond similarities on Shannon index of the farmland, percentage of cropland, amount of fodder distributed per animal and percentage of silage in animal diets (**Figure 8**). Different combinations of farm initial farm configurations and technical adaptations led to similar levels of vulnerability. Among the most vulnerable farms (B2, B1, and B6), B1 had the most diversified land use. B1 had 13% (mean = ±5% among years, Appendix 1) of its area in permanent pastures vs. 67% (±12%) in grass-based ley pastures and 19% (±5%) in crops. Stocking rate was low (1.01 LU/ha) yet mineral fertilizers were applied by the farmer (29 kg N/ha). More than half (54%) of animal feed was distributed, but this percentage varied (±14%) with climatic variability. Silage represented 31% of distributed feed ±8% according to climatic conditions of each year. Concentrate distribution was stable and limited to 0.61 t DM/LU but this amount was high when considering farm productivity. Farmer B1 developed self-adaptability of his farm configuration through farmland diversity but suffered from limited technical efficiency as illustrated by the inconsistency between stocking rate, mineral fertilization, concentrate distribution per animal and farm productivity.

B2 had one of the least diversified land uses, with 83% (±2%) of farmland in permanent pastures, and small areas of grass-based ley pastures (12 ± 2%) and crops (5 ± 2%). Stocking rate was among the lowest of the farm sample (0.90 LU/ha) yet farmers applied mineral fertilizers (20 kg N/ha). As for B1, concentrate distribution was limited, i.e., 0.57 t DM/LU but remained high when considering farm productivity. Reliance on grazing for animal feeding was higher, which led to only 37% of animal feed being distributed. The farmer adjusted this percentage (±12%) and the percentage of harvests as silage and consequently of silage in animal diets (±8%) according to climatic conditions of each year. Thus, farmer B2 promoted managed adaptability of his farm configuration but as for B1, he suffered from limited technical efficiency due to inconsistency between stocking rate, mineral fertilization, concentrate distribution per animal and farm productivity.

B6 lay between B1 and B2, with 36% (±6%) of its area in permanent pastures vs. 61% (±11%) in grass-based ley pastures and 3% (±5%) in crops. Stocking rate was high and variable across years (1.84 LU/ha ±0.38). Grazing was central in farm organization, with only 35% (±9%) of animal feed being distributed. This farmer adapted the rate of mineral nitrogen fertilization (40 kg N/ha ±25) and the amount of concentrate distributed (0.86 t DM/LU ±23%) over the years. As for B1, farmer B6 promoted managed adaptability of his farm configuration but suffered from efficiency issues similar to B1 and B2.

Similarly, the farms considered the least vulnerable (D8, D7, and M2) displayed differences in farm configurations and adaptations implemented by farmers (**Figure 8**). Farm D8 had 30% of its area dedicated to silage maize production against 13% as natural pastures and 43% as grass-based ley pastures. Stocking rate was high (2.05 LU/ha) and stable across years. To sustain this stocking rate, and try to secure fodder production, the farmer applied 87 kg N/ha/year. In this farm, to uncertainty and risks related to grazing, 83% (±4%) of animal feed was distributed, 80% in the form of silage. Concentrate supplementation (1.24 t DM/LU, i.e., 222 g/kg of milk) was efficient compared to local standards (around 250 g/kg of milk, pers. comm.). Farmer D8 did not implement adaptations over the years, keeping all (surveyed) practices constant despite climatic and economic variability. He rather counted on the technical efficiency and robustness of his farm configuration.

In contrast, farm D7 had 20% of its area dedicated to silage maize production, 29% to permanent pastures and 28% to grass-based ley pastures. Stocking rate was intermediate (1.33 LU/ha). Yet, nitrogen fertilization was high (66 kg N/ha) and stable. The percentage of distributed feed was limited to 70% (±3%) of animal intake including 78.4% of silage. Again, concentrate supplementation (1.34 t DM/LU, i.e., 225 g/kg of milk) was efficient compared to local standards. The main difference compared to D8 was that this farmer reacted to contextual variability and adjusted the stocking rate (±18%) and the duration of animal off-farm grazing (±224%) according to annual conditions. Farmer D7 developed these two options to promote managed adaptability of his farm configuration.

Farm M2 differed from D7 and D8 in that the farmer relied on diversity of his farm configuration to self-regulate the impacts of climatic and economic variability. Farm M2 was a mixed farm with both beef and dairy cattle. This farm had only 12% of its area dedicated to silage maize production but included a wide range of crops (32% of its area), cover crops (1%) and pasture types (21% of permanent pastures, 47% of grass-based ley pastures). A higher rate of concentrate supplementation (1.16 t DM/LU, i.e., 313 g/kg of milk) was another source of security for farm productivity against variability. Stocking rate was high (1.61 LU/ha) and stable across years and fertilization was limited (20 kg N/ha). Like farmer D8, farmer M2 did not implement adaptations over the years but counted on robustness and self-adaptability of his farm configuration.

## Discussion

### Implications for Reducing Vulnerability of Cattle Farms to Climatic and Economic Variability

Interannual variability in climatic conditions and economic conditions had no significant effect on vulnerability variables. This was because differences in initial farm configurations and farmers’ technical adaptations were more discriminant than interannual variability in climatic conditions and input or output prices. Moreover, distribution of farms within the factorial design of the PLS was mainly determined by farms’ initial positions rather than by transitions that occurred during the survey period. This reveals initial differences in farm vulnerability were partly decreased through adaptive capacity, but the technical adaptations implemented did not fully compensate for initial differences. Among the attributes of adaptive capacity identified by Marshall et al. (2014), perception of risk and uncertainty, as well as skills for planning farm configurations, were accordingly more important than the ability to cope with change.

Another reason for this result relates to the farm sample. Farm adaptations implemented during the survey period were rather limited. Most farms remained quite similar to their initial configuration. Thus it was not ideal to illustrate the potential of the proposed method to characterize adaptation pathways that reduce most cattle-farm vulnerability to climatic and economic variability. We are currently applying this method on farms operating a conversion to organic farming that implies implementation of more significant adaptations than that of farms surveys for the purpose of this article. Moreover, our sample of farms combined beef, dairy and mixed farms. Patterns of combinations between farmer practices and economic performance might differ among farm types. For this reason, even when changing economic indicators (e.g., to net cash flow), economic performance was not related to specific practices. This could be the reason why we did not explain economic performance whereas we do explain it on other analysis focused on dairy farms that we are currently conducting.

Despite differences in exposure to climatic and economic variability among sample farms, it emerged that functional diversity of the farmland tended to reduce farm vulnerability to climatic and economic variability, as already observed by Martin and Magne (2015). Farmland diversity was consistent with high percentage of cropland on the farm (to grow maize, immature cereals, grain legumes), high percentage of harvests as silage (to harvest maize and immature cereals) and accordingly high amounts of fodder distributed per animal. Technical efficiency which can be assessed through consistency between stocking rate, mineral fertilization, concentrate distribution per animal and farm productivity was another key determinant of farm vulnerability.

No specific combination integrating all studied farmers’ practices emerged that reduces cattle-farm vulnerability to climatic and economic variability. Instead, we observed that different combinations led to similar levels of vulnerability. In these systems, the practices implemented (stocking rate, input use, etc.) were consistent with the objective of developing the properties targeted (efficiency, robustness, adaptability, etc.), as discussed by Bloksma and Struik (2007). For instance, farm D8 was designed for system efficiency (Dumont et al., 2013) and robustness (Ten Napel et al., 2011) through consistent matching of animal diets to animal feeding requirements, of stocking rate to land-use capacity and management intensity, etc. Farm M2 relied on farmland diversity to promote redundancy in animal feed production and adaptability to impacts of climatic and economic variability, as observed by Martin and Magne (2015).

These results confirm earlier findings (Reidsma et al., 2010; Nicholas and Durham, 2012) showing that farm-level adaptation is required to adapt to variability in the production context (climate, prices, etc.). Depending on farm vulnerability, incremental adaptations, system adaptations (Rickards and Howden, 2012), or transformational adaptations might be needed. Incremental adaptations are extension of actions and behaviors already observed to achieve unchanged objectives despite changing conditions. System adaptation engages in modifying farm subsystems (e.g., herd or land use) and revising actions, behaviors and objectives from known examples. Transformational adaptation involves drastic changes at the whole-farm level and in actions and behaviors with in-depth revision of objectives. Incremental adaptations might be sufficient to ensure sustainability of the least vulnerable farms, which have robustness or resilience against climatic and economic variability. In contrast, in the most vulnerable farms, system and transformational adaptation appear to be the only way to improve conditions and ensure their sustainability. It has been shown that capacity to transform a farm varies greatly among farmers (Marshall et al., 2014).

### Merits and Limits of the Method

Longitudinal analysis is a complex and multistage process that can be performed using several approaches. Most scientific work on vulnerability and related concepts including resilience (Darnhofer et al., 2010b) and adaptation (Füssel, 2007) has produced theoretical frameworks and qualitative methods. The integrated and operational method we developed enables assessing farm vulnerability to multiple contextual changes (i.e., both climatic and economic) and explains how this vulnerability can be reduced according to farm configurations and farmers’ technical adaptations over time. Relying on random regressions, it considers farm effects as random effects, which ensures that inferences will be valid for all existing farms. Our methods can be complemented by additional statistical analysis including (i) heatmaps (graphical representation of data where the individual values contained in a matrix are represented as colors) to shed light on farms displaying similar patterns of explanatory and vulnerability variables, and (ii) explanatory variable selection in high dimensional linear mixed model (Rohart, 2016). The latter enables to deepen the relations between a given vulnerability variable (while PLS regression analyzes all the vulnerability variables together) and most robust explanatory variables to predict this variable.

In the area of field-based vulnerability assessments applied to agricultural systems, the integrated method we developed is original in several aspects:

(i) Farm vulnerability is often reduced to productivity or income issues (Reidsma et al., 2010; Dong et al., 2015) and based on raw measurements, while our method enables several vulnerability variables to be addressed. Thus, it is possible to integrate key issues into sustainable agriculture, such as technical and economic efficiency. While other approaches are limited to trends in these variables (Dong et al., 2015), our method breaks them down into mean level, trend, and variability, which enriches the vulnerability assessment. This makes it possible to analyze trade-offs among vulnerability variables and to identify sources of improvements at individual and farm-sample levels. To verify the consistency of the analysis and to exemplify the results, it is often necessary to come back to the individual farm level. This was illustrated by the study presented in this article where the size of the farm sample is limited.

(ii) Unlike previously published quantitative methodologies for assessing farm vulnerability (Reidsma et al., 2009; Dong et al., 2015), our method allows flexibility in the type of vulnerability variables considered (raw measurements, fitted values, slope and residuals of a linear regression; **Figure 2**). For instance, we analyzed our case study using either raw measurements or fitted values. Results of both were quite similar; therefore, we presented only results of raw measurements, but those of fitted values would be more relevant when de-noised data is needed. If fitted values are preferred, the intercept of the corresponding linear regression can replace the mean level. One could also consider a more complex function (e.g., non-linear) instead of a linear mixed model as a straightforward extension. In the study presented in this article, the low number of data per farm over time limited the choice to a linear trend, in addition to the ease of interpretation of this parameter. Moreover, the selected vulnerability and explanatory variables are not a rigid list. Instead, it is possible to fully adapt the list depending on the local context to include vulnerability variables representing, e.g., farmer workload and explanatory variables representing, e.g., farmer knowledge and experience.

Thus, our method does not force users to use a given set of vulnerability variables, remaining adaptable to the specific characteristics of each project.

(iii) Whereas many studies are limited to a single contextual change, mainly climate (Reidsma et al., 2009; Dong et al., 2015), and therefore reduce the complexity of farmers’ decision-making contexts, our method enables addressing several variables that reflect exposure to contextual change, i.e., climatic and economic variability as well as other relevant variables. Moreover, by considering farm effects as individual effects, linear models at the core of our method integrate farm-specific contextual changes not explicitly included in the analysis. Indeed, individual effects result from a set of issues (e.g., family issues, pest outbreaks) hard to characterize with simple indicators or costly to measure.

(iv) Many studies focus on strategic changes and consequently use multi-year time steps [5 years in Rueff et al. (2012), 14 years in García-Martínez et al. (2009)] or provide limited descriptions of farmers’ management practices over time (Reidsma et al., 2010). Our method can consider dynamic changes in these practices and their interactions (i.e., co-occurrence of changes in farmers’ practices) among years. It is even possible to integrate delayed effects of technical adaptations by integrating land use and herd management variables of the previous year as explanatory variables for a given year. For instance, we analyzed our case study with farmers’ practices of each year and of each previous year used as explanatory variables. Results were not changed because technical adaptations implemented by farmers were limited. However, if analyzing farm data over longer time frames (e.g., 10 years), it is possible to characterize delayed effects of past technical adaptations (e.g., 5 years before). Thus, our method considers both farmers’ planning (designing consistent farm layouts) and coping abilities (finding optimal technical adaptations).

(v) Whereas vulnerability assessment methods are not always oriented toward practice (Reidsma et al., 2010; Dong et al., 2015), our method is intended to support agricultural extension agents and farmers in the adaptation process. It provides relevant information about farm vulnerability to climatic and economic variability and about the most promising adaptation pathways. It is currently used in other research and development projects, which enabled us to confirm its ability to understand the drivers of vulnerability within samples of similar farms. For instance, from a France-wide database of organic dairy farms, the method revealed that concentrate feeding was one of the most discriminant practices in a subsample of farms that had more than 90% of their area in grasslands.

## Conclusion

Our method offers insight to unravel the complex issue of cattle-farm vulnerability to climatic and economic variability. It showed that even in a small region such as Aveyron, no single combination of farmers’ practices decreases cattle-farm vulnerability to climatic and economic variability. It also revealed that system and transformational adaptations of the practices implemented (stocking rate, input use, etc.) appears the only way to ensure the sustainability of the most vulnerable farms. Since it can be easily applied to other farming contexts, our method has the potential to support farmers in the development of sector-specific and locally relevant adaptations.

## Author Contributions

GM and M-AM designed the research, supervised data collection, developed the methodology, performed the analysis and wrote the manuscript. Both of them contributed equally. MC supported the development of the method as well as data analysis and interpretation. She also reviewed the manuscript.

## Conflict of Interest Statement

The authors declare that the research was conducted in the absence of any commercial or financial relationships that could be construed as a potential conflict of interest.
