# Supplementary material for: An Integrated Method to Analyze Farm Vulnerability to Climatic and Economic Variability According to Farm Configurations and Farmers’ Adaptations
Source: Front Plant Sci. 2017 Aug 29;8:1483. doi: 10.3389/fpls.2017.01483 (PMC5581829; doi:10.3389/fpls.2017.01483)
Supplement: Supplementary file 1 [file Table_1.docx]

Appendix 1: Mean and standard deviation of explanatory variables related to vulnerability variables on the two first components of the PLS regression

|  | ShannonLand | | %Cropland | | %NatPast | | %GrassPast | | %CoverCrop | | NminFert | | IrrigWater | | StockingRate | | %SilageFeed | | FodderDistrib | | ConcDistrib | | CalvingSpread | |
| --- | --- | --- | --- | --- | --- | --- | --- | --- | --- | --- | --- | --- | --- | --- | --- | --- | --- | --- | --- | --- | --- | --- | --- | --- |
| Farm | mean | sd | mean | sd | mean | sd | mean | sd | mean | sd | mean | sd | mean | sd | mean | sd | mean | sd | mean | sd | mean | sd | mean | sd |
| B1 | 1,24 | 0,25 | 18,7 | 5,5 | 12,6 | 5,3 | 67,1 | 12,2 | 0,0 | 0,0 | 29 | 10 | 0 | 0 | 1,01 | 0,14 | 30,6 | 8,0 | 2,58 | 0,67 | 0,61 | 0,05 | 0,14 | 0,02 |
| B10 | 1,99 | 0,06 | 20,5 | 0,7 | 26,9 | 1,0 | 52,4 | 1,3 | 0,0 | 0,0 | 26 | 8 | 0 | 0 | 1,50 | 0,03 | 22,8 | 5,1 | 1,74 | 0,53 | 1,10 | 0,12 | 0,09 | 0,00 |
| B2 | 0,81 | 0,07 | 5,2 | 1,6 | 82,8 | 2,4 | 12,0 | 2,4 | 0,0 | 0,0 | 20 | 3 | 0 | 0 | 0,90 | 0,14 | 13,4 | 7,7 | 1,78 | 0,56 | 0,57 | 0,11 | 0,12 | 0,03 |
| B3 | 0,39 | 0,54 | 0,0 | 0,0 | 100,0 | 0,0 | 0,0 | 0,0 | 0,0 | 0,0 | 0 | 0 | 0 | 0 | 0,86 | 0,04 | 0,9 | 1,3 | 2,37 | 0,38 | 0,40 | 0,32 | 0,19 | 0,04 |
| B6 | 1,06 | 0,23 | 3,4 | 5,2 | 35,7 | 6,4 | 61,0 | 10,6 | 0,0 | 0,0 | 40 | 25 | 0 | 0 | 1,84 | 0,38 | 53,8 | 10,7 | 1,66 | 0,44 | 0,86 | 0,19 | 0,09 | 0,01 |
| B7 | 1,14 | 0,13 | 4,9 | 2,5 | 31,8 | 2,0 | 63,4 | 3,6 | 0,0 | 0,0 | 21 | 12 | 0 | 0 | 1,12 | 0,08 | 0,0 | 0,0 | 1,82 | 0,32 | 0,41 | 0,05 | 0,20 | 0,03 |
| B8 | 1,85 | 0,16 | 17,8 | 5,2 | 24,8 | 8,9 | 46,9 | 4,5 | 2,1 | 2,2 | 80 | 12 | 0 | 0 | 1,68 | 0,24 | 76,6 | 12,7 | 2,65 | 0,33 | 1,03 | 0,08 | 0,08 | 0,00 |
| B9 | 2,17 | 0,04 | 27,0 | 4,5 | 22,0 | 3,2 | 51,1 | 4,6 | 8,3 | 2,9 | 70 | 10 | 4582 | 1934 | 2,17 | 0,06 | 84,3 | 5,5 | 1,96 | 0,25 | 0,80 | 0,10 | 0,08 | 0,00 |
| D1 | 1,79 | 0,06 | 23,7 | 9,4 | 26,0 | 2,8 | 46,8 | 4,5 | 7,3 | 8,2 | 29 | 32 | 10858 | 9192 | 1,26 | 0,12 | 76,1 | 5,8 | 3,63 | 0,44 | 0,89 | 0,22 | 0,09 | 0,00 |
| D10 | 1,84 | 0,05 | 29,7 | 3,2 | 34,0 | 3,5 | 36,4 | 2,6 | 0,0 | 0,0 | 53 | 15 | 2125 | 3301 | 1,27 | 0,08 | 78,6 | 7,0 | 3,15 | 0,92 | 1,09 | 0,20 | 0,09 | 0,01 |
| D11 | 2,20 | 0,16 | 33,8 | 2,3 | 39,4 | 0,1 | 26,9 | 2,3 | 2,9 | 2,9 | 70 | 5 | 0 | 0 | 1,38 | 0,08 | 70,6 | 4,8 | 3,44 | 0,33 | 1,38 | 0,09 | 0,12 | 0,01 |
| D2 | 1,38 | 0,49 | 16,8 | 1,0 | 18,6 | 10,0 | 63,8 | 10,0 | 4,6 | 6,4 | 53 | 30 | 0 | 0 | 1,10 | 0,05 | 2,8 | 2,6 | 3,42 | 1,15 | 1,32 | 0,20 | 0,12 | 0,02 |
| D3 | 2,36 | 0,15 | 31,0 | 3,2 | 37,0 | 5,6 | 23,4 | 11,6 | 10,8 | 1,3 | 40 | 8 | 23039 | 16519 | 0,72 | 0,03 | 61,6 | 6,3 | 3,79 | 0,29 | 1,50 | 0,28 | 0,09 | 0,00 |
| D6 | 1,62 | 0,13 | 18,9 | 1,9 | 8,6 | 0,1 | 72,4 | 1,9 | 14,8 | 4,3 | 0 | 0 | 7710 | 3094 | 1,12 | 0,07 | 65,4 | 7,5 | 3,13 | 0,30 | 0,52 | 0,08 | 0,09 | 0,00 |
| D7 | 2,28 | 0,10 | 41,8 | 4,5 | 28,9 | 2,2 | 27,9 | 6,1 | 0,8 | 1,7 | 66 | 12 | 0 | 0 | 1,33 | 0,24 | 78,4 | 7,0 | 3,31 | 0,15 | 1,34 | 0,20 | 0,09 | 0,00 |
| D8 | 1,97 | 0,13 | 42,4 | 4,8 | 13,3 | 0,0 | 42,7 | 5,0 | 14,3 | 4,4 | 87 | 6 | 0 | 0 | 2,05 | 0,15 | 80,1 | 4,9 | 3,95 | 0,20 | 1,24 | 0,20 | 0,13 | 0,01 |
| D9 | 1,60 | 0,03 | 38,1 | 1,5 | 9,9 | 2,4 | 52,0 | 1,3 | 1,7 | 3,5 | 73 | 6 | 0 | 0 | 1,59 | 0,12 | 78,3 | 9,4 | 4,23 | 1,84 | 2,15 | 0,25 | 0,09 | 0,00 |
| M2 | 1,92 | 0,12 | 31,9 | 3,8 | 20,7 | 0,5 | 46,9 | 3,2 | 0,7 | 1,6 | 20 | 12 | 0 | 0 | 1,61 | 0,04 | 76,9 | 5,8 | 3,22 | 0,32 | 1,16 | 0,27 | 0,11 | 0,01 |
| M3 | 2,38 | 0,04 | 33,6 | 2,2 | 34,7 | 0,0 | 30,2 | 3,1 | 9,5 | 3,1 | 72 | 5 | 7410 | 1766 | 1,45 | 0,07 | 72,0 | 4,2 | 3,71 | 0,38 | 1,28 | 0,15 | 0,09 | 0,00 |
